# DNA micelle flares: a study of the basic properties that contribute to enhanced stability and binding affinity in complex biological systems†
†Electronic supplementary information (ESI) available. See DOI: 10.1039/c6sc00066e


**DOI:** 10.1039/c6sc00066e

**Published:** 2016-05-23

**Authors:** Yanyue Wang, Cuichen Wu, Tao Chen, Hao Sun, Sena Cansiz, Liqin Zhang, Cheng Cui, Weijia Hou, Yuan Wu, Shuo Wan, Ren Cai, Yuan Liu, Brent S. Sumerlin, Xiaobing Zhang, Weihong Tan

**Affiliations:** a Center for Research at Bio/Nano Interface , Department of Chemistry , Department of Physiology and Functional Genomics , Health Cancer Center , UF Genetics Institute and McKnight Brain Institute , University of Florida , Gainesville , Florida 32611-7200 , USA . Email: tan@chem.ufl.edu; b Molecular Science and Biomedicine Laboratory , State Key Laboratory for Chemo/Bio-Sensing and Chemometrics , College of Chemistry and Chemical Engineering , College of Biology , Collaborative Research Center of Molecular Engineering for Theranostics , Hunan University , Changsha 410082 , China; c George & Josephine Butler Polymer Research Laboratory , Center for Macromolecular Science & Engineering , Department of Chemistry , University of Florida , Gainesville , Florida 32611-7200 , USA

## Abstract

DMFs are spherical DNA–diacyllipid nanostructures formed by hydrophobic effects between lipid tails coupled to single-stranded DNAs. Such properties as high cellular permeability, low critical micelle concentration (CMC) and facile fabrication facilitate intracellular imaging and drug delivery. While the basic properties of NFs have been amply described and tested, few studies have characterized the fundamental properties of DMFs with particular respect to aggregation number, dissociation constant and biostability. Therefore, to further explore their conformational features and enhanced stability in complex biological systems, we herein report a series of characterization studies. Static light scattering (SLS) demonstrated that DMFs possess greater DNA loading capacity when compared to other DNA-based nanostructures. Upon binding to complementary DNA (cDNA), DMFs showed excellent dissociation constants (*K*_d_) and increased melting temperatures, as well as constant CMC (10 nM) independent of DNA length. DMFs also present significantly enhanced stability in aqueous solution with nuclease and cell lysate. These properties make DMFs ideal for versatile applications in bioanalysis and theranostics studies.

## Abstract 2

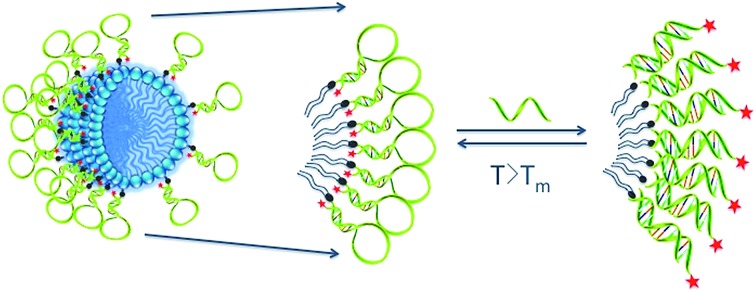
DMFs are spherical DNA–diacyllipid nanostructures formed by hydrophobic effects between lipid tails coupled to single-stranded DNAs.

## Introduction

The hydrophobic effect of DNA-related amphiphiles has recently attracted considerable attention in the areas of bioanalysis and biomedicine. Spherical structures, such as spherical nucleic acids (SNAs) and DNA block copolymers (DBCs) are significant forerunners in the detection and treatment of cancer cells, and, more importantly, the generation of many other innovative structures. DBCs are oligonucleotides coupled with hydrophobic polymers that can self-assemble into three-dimensional micelle nanostructures. Compared to other biopolymer analogues, DBCs have such advantages as nucleotide precision and controllable supramolecular structure, leading to applications ranging from biomarker discovery to drug delivery.^1^,^2^ In aqueous solution, DBCs self-assemble as a result of microphase separation, while a reversible hydrophobic effect keeps the polymeric chains together.^3^ This uniquely dynamic micelle structure has proven particularly useful as both carrier for anticancer drugs and scaffold for specific chemical reactions.^4^,^5^ For example, in the DBC micelle-based drug delivery system, a hydrophobic anticancer drug was loaded into the DBC micelle core, and further functionalization was achieved through folic acid-modified DNA strands complementary to DNA sequences on the surface of the DBC micelles.^6^

Despite many successful applications, DBCs still have such disadvantages as complicated preparation and high critical micelle concentrations (CMC). To simplify preparation and achieve maximum assembly efficiency, our group reported a DNA micelle flare nanostructure consisting of a diacyllipid core and a single-stranded DNA corona.^7^ DMFs share many unique properties with DBCs, including the ability to self-assemble through the hydrophobic effect and carry oligonucleotides outside, as well as the capacity to be doped with certain hydrophobic dyes or drugs. However, DMFs can be further characterized by their outstanding cell permeability, which is facilitated by the similarity between the intermolecular forces of the diacyllipid in DNA micelle flares and those of the dynamic phospholipid bilayers in cell membranes, both of which have hydrophobic and hydrophilic portions. Here, strong van der Waals forces result in efficient transport of antisense oligonucleotides and drug molecules to live cells.^8^–^10^ For example, hairpin-shaped DMFs successfully entered living cells and emitted fluorescent signals upon binding to specific target molecules as a result of opening of the hairpin and increasing the distance between the fluorophore and quencher (Fig. S2†).^11^ When incorporated with a targeting aptamer, DNA micelle flares can selectively recognize specific types of cancer cells.^7^ Thus, with their combined cell internalization and targeting capabilities, aptamer-micelles can find many applications in biomedicine, including drug delivery *via* endocytosis and gene therapy through the inhibition of cancer-related mRNA expression.^11^,^12^

To undertake a study of the basic properties associated with DNA micelle flares, one may look to the pioneering work of Mirkin *et al.* for guidance. For the sensitive detection of mRNA in living cells, nanoflares (NFs) “exhibit high signaling, have low background fluorescence, and are sensitive to changes in the number of RNA transcripts present in cells”.^13^ Similar to DMFs, nanoflares present densely functionalized DNA on the surface of gold nanoparticles (NPs). NFs also have significant chemical and physical advantages that are distinct from DNA and NPs, enabling them to act as scaffolding for the self-assembly of oligonucleotides. The gold core of nanoflares can be dissolved after fabrication to make hollow spherical nucleic acids (SNAs). These SNAs show stronger complementary nucleic acid hybridization and more efficient cell membrane transfection than NFs having gold cores.^14^ Moreover, the properties of NFs have been systematically investigated, including measurement of melting transition, binding strength and biostability. The results have inspired the innovative design of many bioprogrammable nanostructures that have become potential tools in biomedicine and areas related to energy conversion.

**Scheme 1:**
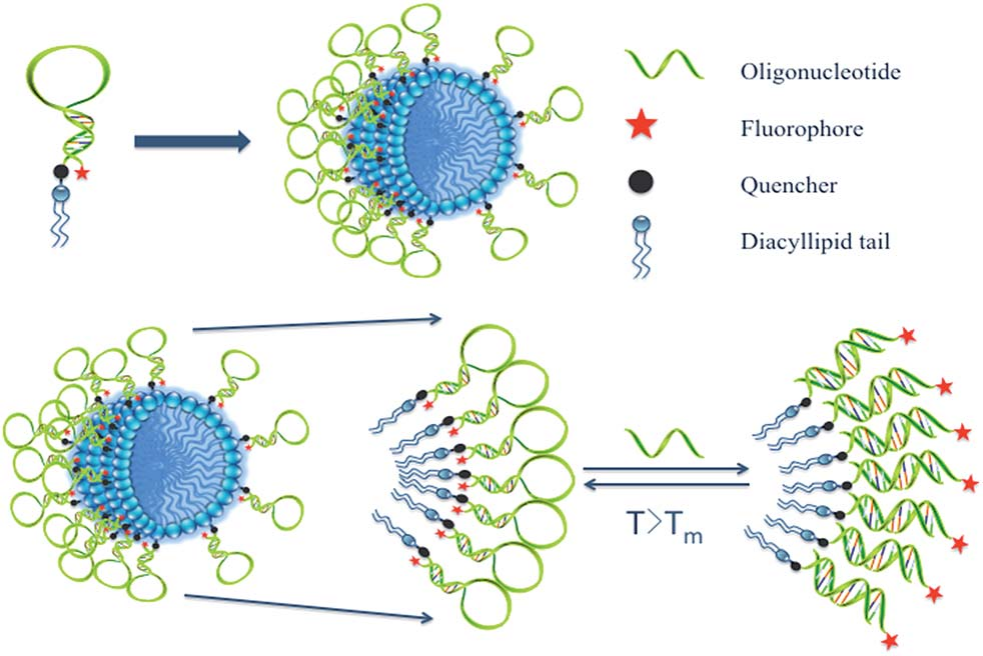
Illustration of a molecular beacon micelle flare nanostructure. Hairpin-shaped DNA–diacyllipid conjugates self-assemble into a spherical micelle flare nanostructure, in which the hairpin-shaped DNA corona can lead to an ON/OFF transition upon target binding, temperature change or degradation.

Inspired by the extensive studies of NFs, the present work investigated the properties of DNA micelle flares that lead to enhanced stability and binding ability. These structures are comprised of either plain ssDNA or hairpin-shaped (molecular beacon) segments. In particular, molecular beacon micelle flares (MBMFs), which are easily prepared through diacyllipid phosphoramidite chemistry using an automated DNA synthesizer, undergo a significant fluorescence signal change when binding to targets.^12^ In the absence of target cDNA, MBMFs exhibit very low fluorescence because the fluorophore and quencher are spatially close. However, upon binding to target DNA, fluorophore and quencher are separated, resulting in the restoration of fluorescence signal (Scheme 1). In this work, studies of complementary binding assays and biostability are based on this concept. In complex biological media, such as nuclease-containing systems or cell lysate, this structure offers resistance towards enzymatic digestion and maintains its size, suggesting the utility of this tool in cell detection, gene silencing and drug delivery.

## Results and discussion

### Compositional study

MBMFs must first hybridize with mRNA as a precondition for use in gene therapy. The probe then acts either by blocking translation of the targeted mRNA or by forming a DNA/RNA hybrid with the target mRNA. Either mRNA or the DNA/RNA hybrid can be degraded by the enzyme RNase H. Using these mechanisms, MBMFs can be used for imaging guided gene therapy.^15^ Therefore, as a potential tool for cancer gene therapy, increasing the DNA loading capacity of micelle flares would greatly improve therapeutic effect based on the interaction between micelle flares and intracellular cancer-related mRNAs. As such, an essential clinical parameter is aggregation number (*N*_agg_), *i.e.*, the number of monomers present in a micelle flare. Previous research has shown that the sizes and aggregation behavior of polymers can be measured with dynamic light scattering (DLS) and static light scattering (SLS), respectively,^1^,^15^ including measurements related to diffusion coefficients (*D*), hydrodynamic radius (*R*_h_) and aggregation phenomena of molecules in aqueous solution. Therefore DLS/SLS offers the most straightforward technique for the study of intramicelle interactions in aqueous solution.^16^

In this study, DMFs with 60-thymine base oligonucleotide sequences and a diacyllipid tail (lipo-T_60_) were characterized. First, the number-average apparent molecular weight (*M*_n,RI_) of a micelle monomer, diacyllipid–T_60_ conjugate, was determined using polyethylene glycol (PEG) standards in aqueous size exclusion chromatography (SEC). The observed *M*_n,RI_ was 1.25 × 10^4^ g mol^–1^, which was lower than the theoretical molecular weight (*M*_n,theory_) of 18 941 g mol^–1^. This low molecular weight value can be attributed to the highly compact DNA self-assembled nanostructure, leading to a small hydrodynamic radius. For better accuracy, we chose the observed *M*_n,RI_ for further calculation, and multi-angle light scattering (MALS) was used to measure the scattered light intensity at various detection angles. ASTRA 6 software was used to evaluate the absolute molecular weight of the molecule (Fig. S1†). The absolute number-average molecular weight (*M*_n,SLS_) for a micelle in a 20/80 mixture of acetonitrile/0.05 M Na_2_SO_4_ was found to be 6.8 × 10^6^ g mol^–1^, according to SLS-SEC measurements. This result confirmed that the proposed architecture results from a large number of self-assembled DNA–diacyllipid amphiphiles.

Next, the average aggregation number for the DNA micelles was calculated from the measured molecular weights according to eqn (1)1
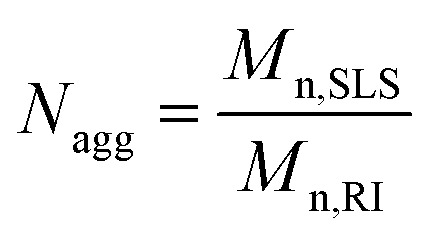
to give 6.8 × 10^6^/1.25 × 10^4^ = 544 for lipo-T_60_. This number corresponds to the maximum DNA loading of DNA nanoflares with minimal addition of linkers and ions.^17^

**Fig. 1:**
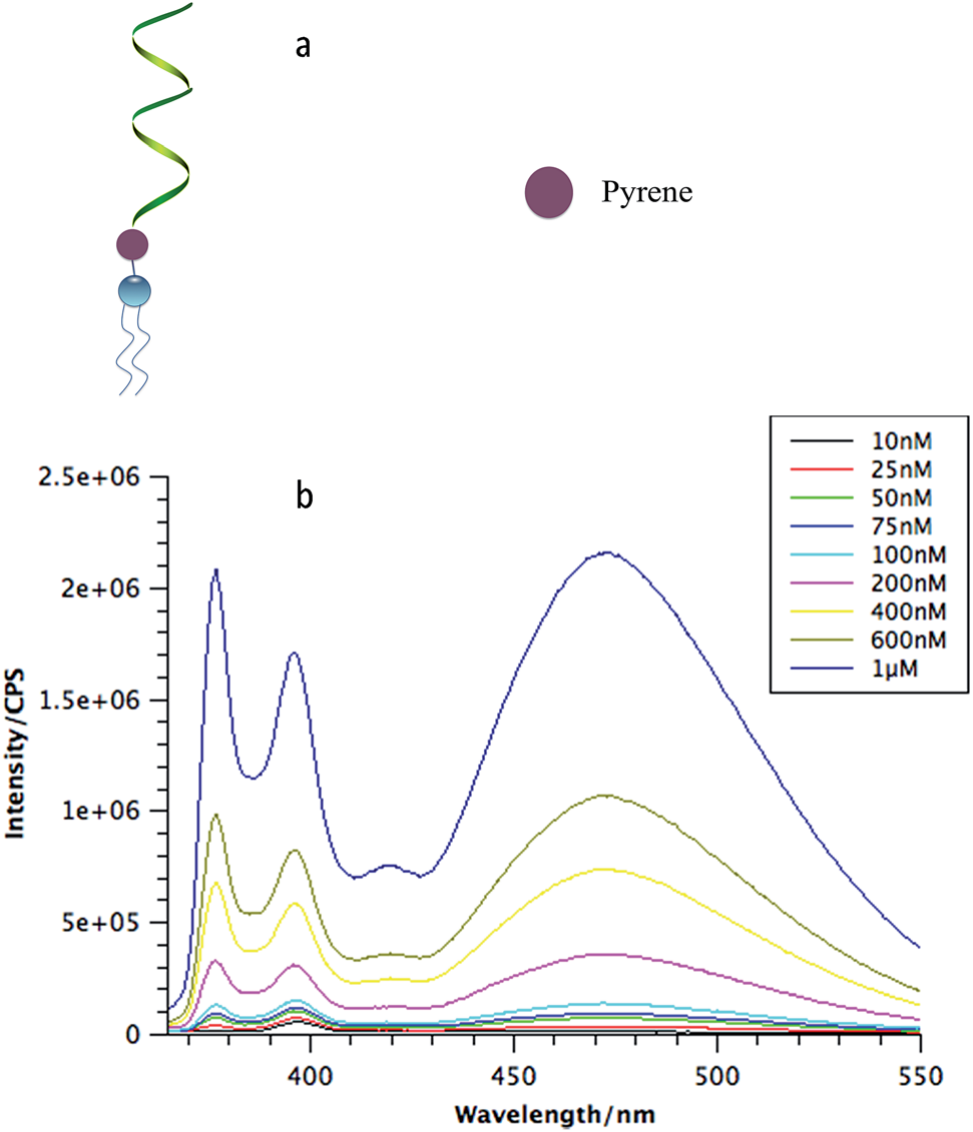
(a) The structure of a DNA micelle monomer that has a pyrene molecule coupled between the single-stranded DNA and lipid tail; all the monomers were coupled with pyrene; (b) CMC study of DNA micelle flares with 60-thymine bases. DNA micelle flares were diluted to different concentrations in PBS, followed by measuring the fluorescence intensity of pyrene excimer (ex: 335 nm, em: 470 nm).

To analyze the biostability of DNA micelle flares, a key parameter is the critical micelle concentration, which is defined as the concentration of monomers above which DNA micelle flares form and all additional monomers added to the system go to micelles. Here, a unique fluorophore, pyrene, was incorporated between the DNA corona and the lipid core (Fig. 1a). A pyrene unit is often used to probe aggregation behavior in various conditions based on its special fluorescence characteristics.^18^ Specifically, when monomers aggregate, multiple pyrene molecules will cluster together and generate an excimer-type fluorescence signal with larger Stokes shift.^19^

To determine the CMC of DNA micelle flares, three different lengths of DNA strands with 20, 40 and 60 thymine bases (lipo-T_20_, lipo-T_40_, and lipo-T_60_) were synthesized and modified with pyrene and diacyllipid, respectively. The micelle flares were then incubated in phosphate-buffered saline (PBS) and characterized using steady-state fluorescence spectroscopy. As shown in Fig. 1b, the lipo-T_60_ micelle flares had a very low CMC value (about 10 nM). The fluorescence intensities have also been plotted against the concentration of DMFs and a linear fit of the signal has been observed (Fig. S4c†). Further study of lipo-T_20_ and lipo-T_40_ showed CMC values similar to that of lipo-T_60_, indicating that the formation of DNA micelle flares is size-independent (Fig. S4a and b†).^1^

The CMC is a key parameter characterizing surfactants and, hence, is unrelated to hydrophilic DNA length based on the identical diacyllipid core, which accounts for the aggregative nature of DNA micelle flares. Nevertheless, this low CMC does suggest the greater stability of DMFs, even in diluted solution or *in vivo* environments, when used for cancer treatment.

### Enhanced binding affinity of DNA micelle flares

**Fig. 2:**
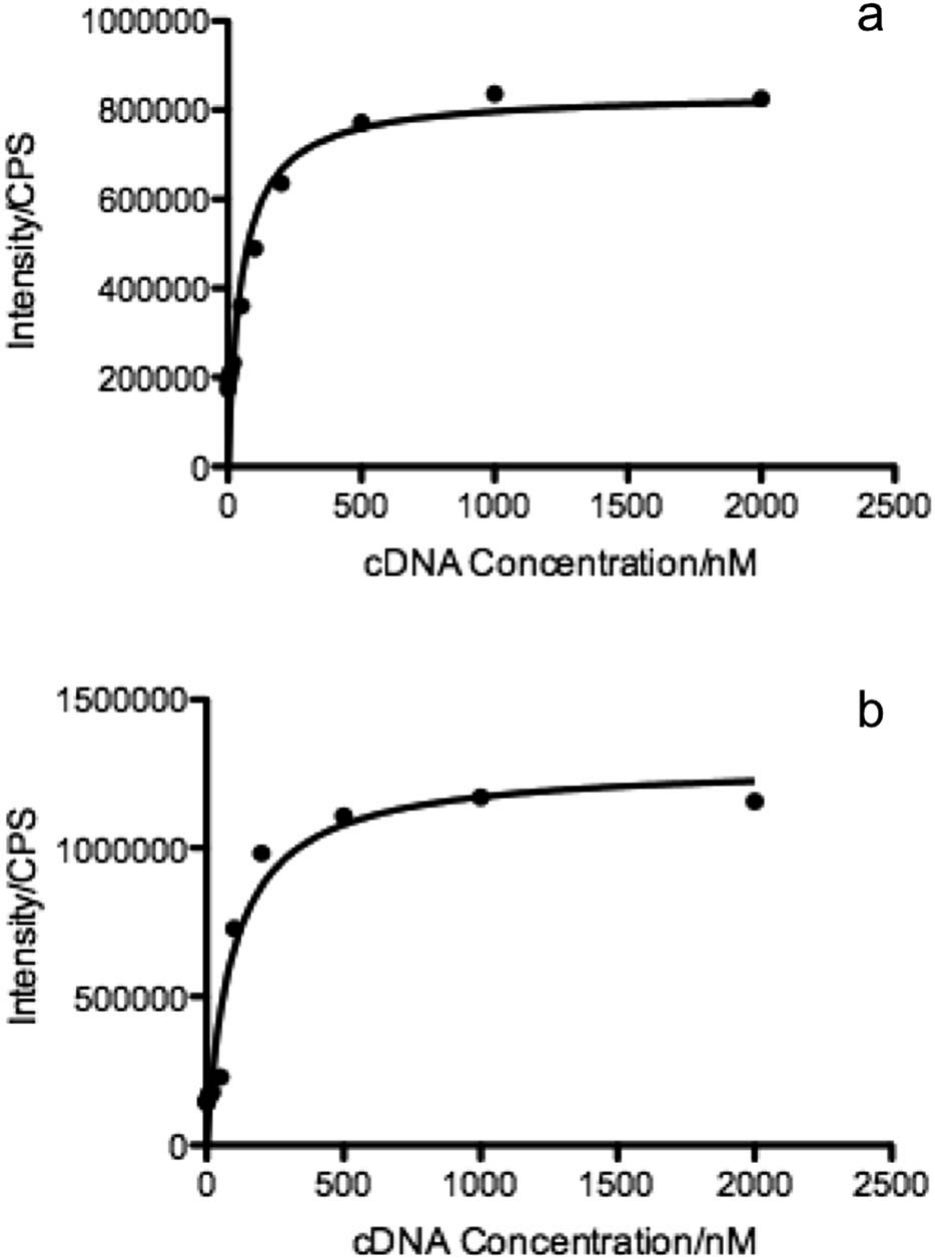
Comparison of binding affinity between MBMF and MB. The dissociation constants of (a) MBMFs, *K*_d_ = 52 nM, and (b) MBs, *K*_d_ = 95 nM. MBMFs and MBs were titrated with various concentrations of cDNA in PBS, followed by measuring their fluorescence intensities. Dissociation constant curves were plotted according to the fluorescence intensity and the concentration of cDNA.

The molecular recognition of nucleic acids to target DNA/RNA provides sensitive detection and promotes gene silencing, both useful in cancer theranostics. As such, the binding affinity of DMFs to target was compared to that of the DNA probe without diacyllipid conjugation. In this experiment, TAMRA (carboxytetramethylrhodamine) and Dabcyl (dimethylaminoazobenzenesulfonic acid) were chosen as fluorophore and quencher, respectively, and coupled on the ends of the hairpin-shaped DNA. To evaluate the dissociation constant (*K*_d_) of MBMFs, we compared the binding affinity of MBMFs and molecular beacons (MBs) with the same target, the DNA analogue of partial c-raf-1 mRNA, a cancer biomarker and antisense therapeutic target. The fluorescence titration assay was used to quantify the dissociation constant with increased concentrations of target DNA. Opening of the hairpin structure resulted in the separation of TAMRA and Dabcyl, which, in turn, caused a change in peak intensity, thus forming the basis of our measurement. GraphPad Prism 5.0 software was used to construct a binding isotherm, and *K*_d_ was calculated by analyzing the binding curve according to eqn (2)^20^2
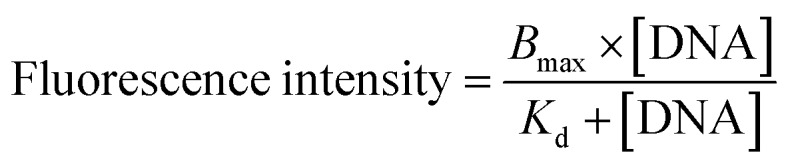
where *B*_max_ stands for the binding affinity at maximum fluorescence intensity. As shown in Fig. 2, the *K*_d_s of MBMFs and MBs were calculated to be 52 and 95 nM, respectively, demonstrating that MBMFs have stronger binding affinities with their cDNA compared to MBs. This phenomenon can be explained by the density of DNA oligonucleotides on the surface of DNA micelles, which makes it difficult for cDNA to dissociate from the probes. This will greatly lower the cellular detection limit in that it gives a considerable level of signal, even for mRNA with low-expression level, thus improving the efficiency of gene therapy when antisense DNA micelles are being used.

The addition of heat to the hybridization complex between molecular beacons and their target cDNAs breaks the hydrogen bonds between base pairs, resulting in dissociation of the complexes and restoration of the hairpin structure. Thus, a decrease of fluorescence signal is observed by the resumption of the quenching effect, as shown in Scheme 1, when the system reaches the melting temperature (*T*_m_). Here, the melting temperatures were quantified using a real-time polymerase chain reaction (qPCR) instrument that can monitor the unfolding procedure of the hairpin structures for each 1 °C increment by labeling each DNA probe with a fluorophore and a quencher. In this assay, MBMF-cDNA and MB-cDNA at the same concentration were heated, followed by monitoring the fluorescence intensity.^21^ The temperature range of 60 to 80 °C was chosen according to the theoretical *T*_m_ of oligonucleotide hybridization, 69.7 °C.

**Fig. 3:**
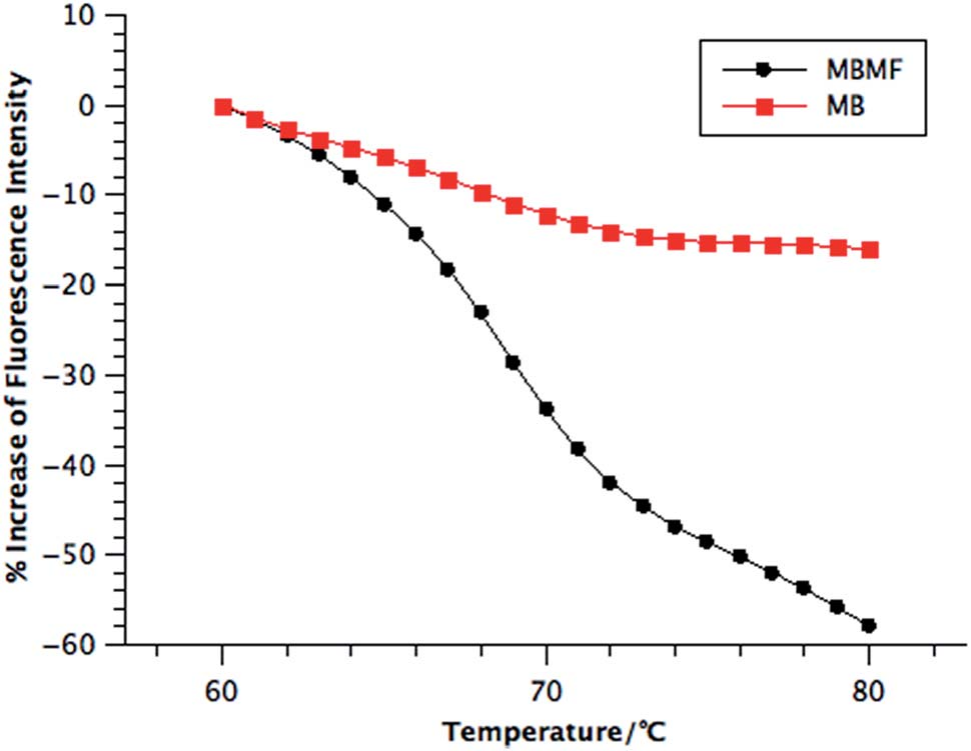
Melting transition of MBMFs and MBs. Probes and cDNA were both diluted to 200 nM and incubated in PBS followed by qPCR. The melting temperatures of MBMFs and MBs with cDNA were 69.0 °C and 68.0 °C, respectively.

Compared to MBs, MBMFs exhibited a sharper transition and slightly higher melting temperature, as shown in Fig. 3. The sharpness most likely results from the cooperative binding of oligonucleotides, while the higher melting temperature occurs as a result of enhanced interparticle connections endowed by the higher density of oligonucleotides on the surface of micelle flares.^13^ Since MBMFs can reach greater effective concentration, they can give a dramatically decreased fluorescence signal. By observing the change of fluorescence in both situations and determining the point where the slope reached a peak value, the *T*_m_s were calculated to be 69 °C for MBMFs and 68 °C for MBs. Like nanoflares, DNA micelle flares exhibit sharper melting temperature increases with their cDNA targets compared to MBs, which implies an enhanced binding affinity and excellent thermal stability (Fig. S5†).^21^

### Biostability of DNA micelle flares in biological environments

**Fig. 4:**
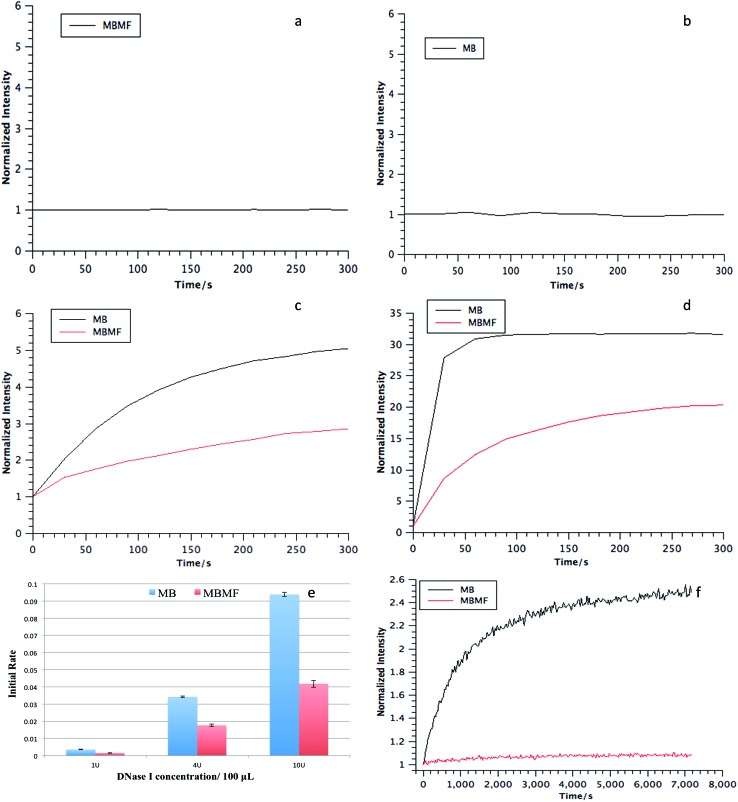
(a and b) Fluorescence signal of MBMFs and MBs when incubated in PBS buffer. (c and d) Enzymatic resistance of MBMFs towards nucleases. MBMFs showed greater resistance than MBs in the presence of (c) DNase I and (d) Exo III. All intensity data were achieved by normalizing the fluorescence signal according to the initial intensity and reaction time. (e) Initial nuclease (DNase I) digestion rates for MBs and MBMFs. MBMFs had a significantly slower initial rate compared to that of MBs for different DNase I concentrations: 1 U, 4 U and 10 U for every 100 μL. Initial reaction rates were calculated using the relative fluorescence change in the first 120 seconds. (f) Fluorescence assay of degradation curves of MBMFs and MBs in CCRF-CEM cell lysate.

As a drug carrier, DMFs must maintain stability in intracellular environments containing various nucleases. Therefore, the nuclease resistance of MBMFs was evaluated relative to that of MBs for two nucleases, deoxyribonuclease I (DNase I) and exonuclease III (Exo III), by monitoring the fluorescence signal change upon the addition of each enzyme. DNase I cleaves DNA in a nonspecific manner, and Exo III primarily recognizes blunt ends or recessed 3′ hydroxyls and digests towards the 5′-end.^22^,^23^ Normally, in an aqueous solution, such as PBS, neither MBMFs nor MBs will have a fluorescence change according to the incubation time (Fig. 4a and b). If the hairpin structure on the surface of the micelle flare is cleaved, no quenching between TAMRA and Dabcyl will occur, leading to an increased fluorescence signal.^24^ As shown in Fig. 4, the intensities were set to 1 after initial addition of nucleases. In contrast to the MBs, the MBMFs exhibited significantly less increase in fluorescence intensity, indicating the enhanced resistance of MBMFs towards both DNase I and Exo III. This phenomenon agrees well with the comparison of nanoflares and MBs, where nanoflares showed a smaller degradation rate.^13^

Initial degradation rates for three concentrations of DNase I are presented in Fig. 4e, showing much lower rates for MBMFs compared to MBs. Thus, MBMFs can maintain the integrity of their nanostructure when treated with nucleases. In addition, the antinuclease property of DMFs was also comparable to that of NFs under the same conditions.^13^ To further confirm that DMFs can maintain their assembled structures, as a key indicator of stability, assays were performed in CCRF-CEM (human T cell lymphoblast-like cell line) cell lysate. Dynamic light scattering data for MBMFs in PBS before and after addition of CEM lysate indicated a hydrodynamic radius of 20 nm, which was in accordance with the length of DNA sequence in micelle flare nanostructures. By comparison to Fig. 4f, it can also be seen that MBMFs presented much higher resistance towards enzymatic digestion. These results clearly showed that DMFs can also maintain their assembled structures in cell lysate, providing an opportunity for their application in cellular and even *in vivo* environments, since DMFs can permeate cell membranes and interact with biomolecules inside cells.^11^

### DMFs, as applied to gene recognition and endocytosis in biological environments

As envisioned by the Tan group, it was found that “the hybridization of MBMFs to the target mRNA can specifically inhibit gene expression through different mechanisms, including translational arrest by steric hindrance of ribosomal activity and the induction of RNase H endonuclease activity leading to the suppression of cancer cell growth,” thus establishing the function of DMFs in gene therapy. However, while MBMFs show strong resistance towards nuclease degradation when compared to MBs, as demonstrated in the present paper, biostability relative to their real-time use in cancer cell recognition and gene therapy has not yet been demonstrated. Therefore, another experiment was designed to confirm the enhanced mRNA binding behavior of MBMFs, and after MBMFs and MBs were individually incubated in CEM lysate for 2 hours, their mRNA binding affinity could be evaluated. Since binding events, which can cause the unfolding of the hairpin structures, may occur only with the addition of cDNA solution, fluorescence spectroscopy is a dependable method for comparing the binding capacity of MBMFs and MBs. A higher fluorescence signal would suggest an enhanced binding affinity resulting from higher concentration of intact structures that were not digested during the 2 hour incubation time.

**Fig. 5:**
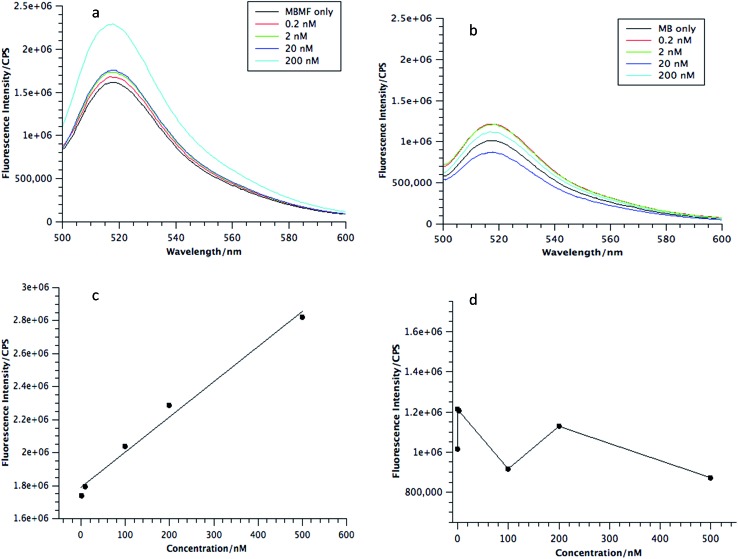
Enhanced cDNA binding of MBMFs. The upper images give the fluorescence emission spectra of the cDNA titrated into (a) MBMF/CEM lysate and (b) MB/CEM lysate solutions. Significant increases were observed in fluorescence signal at 520 nm for MBMFs. The lower images are linear regression curves for the binding with cDNA solutions of (c) MBMFs and (d) MBs, ranging from 0 to 500 nM.

As shown in Fig. 5a and b, by titrating cDNA solution into MBMF or MB/lysate buffer, an increasing fluorescence signal at 580 nm was detected in both systems, indicating the disturbance of the hairpin structure. Compared with the MBs' spectrum, MBMFs could bind with cDNAs in very low concentrations and the fluorescence intensity displayed a stronger cDNA concentration dependency. To further quantify the limits of detection (LODs) of both MBs and MBMFs, fluorescence characterization was carried out using a cDNA concentration gradient (Fig. 5c and d). The LODs were calculated according to eqn (3)3
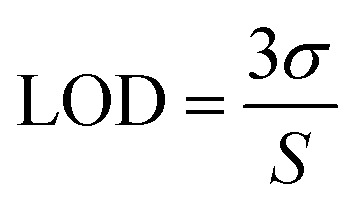
where *σ* is standard deviation of the fluorescence intensity at low cDNA concentration, and *S* is slope of the calibration line. The LOD of cDNA by MBMFs was calculated to be 37 nM, whereas that by MBs could not be calculated. Therefore, when combined with strong resistance to nuclease digestion (Fig. 4f), this proven enhanced cDNA binding affinity further demonstrates the biostability required for practical applications in genetic engineering, *e.g.*, carriers for gene transplantation.

**Fig. 6:**
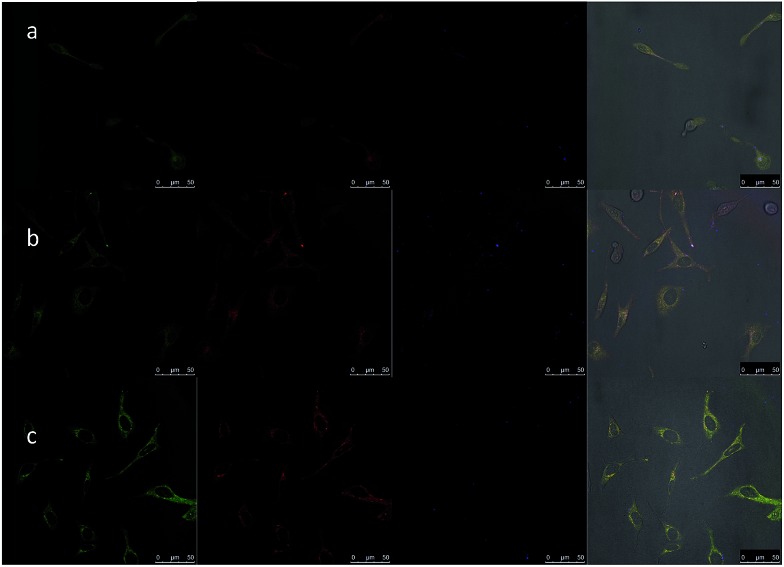
Analysis of endocytosis of DMFs in HeLa cells with incubation times of (a) 30 min, (b) 2 h and (c) 4 h. HeLa cells were cultured in confocal dishes 6–8 h prior to characterization in order to optimize the number of cells. Fluorescence signals increased according to the increment of incubation time, illustrating the progress of probes as they successfully entered the entire cell. The scale bar is 50 μm.

Defining cancer cell endocytosis pathways of DMFs would be beneficial for analyzing cell fusion capability and maximizing oligonucleotide carrying efficiency of the structure. In the absence of such study, the effect could be predicted from time-dependent imaging of fluorescent library DMFs with HeLa cells. First, an MTS-assay was carried out to prove the bio-compatibility of DMFs. The cell viability of HeLa cells with DMF concentrations from 50 nM to 10 μM was characterized and an average of above 90% was observed (Fig. S6a†). This endocytosis experiment used confocal microscopy and organelle dyes, including LysoSensor Green and transferrin-alexa633 (Fig. 6). LysoSensor is a fluorescent dye for lysosomes and transferrin has been widely used to identify the location of endosomes. In this study, as incubation time increased from 30 min to 4 h, the probes gradually escaped from endosomes and entered most of the lysosomes, and finally entered the whole cell except for the nucleus. While the fluorescence signals of TAMRA-labeled DMFs (red), LysoSensor (green) and transferrin (blue) overlapped, most red signals were observed inside the cells, suggesting that the major portion of the probes stayed in the cytoplasm. This result shows that DMFs can serve as gene carriers for certain molecular beacons and DNAzymes. On the contrary, for normal fluorescence-labeled DNAs, there is minimal cellular entry after a 2 h incubation (Fig. S6b and c†).

## Experimental

### DNA synthesis

All oligonucleotides were synthesized by an automated ABI 3400 DNA synthesizer (Applied Biosystems, Inc, Foster City, CA) at 1.0 μM scale. Diacyllipid phosphoramidite was then coupled to the oligonucleotides using the same instrument. Micelles with TAMRA modification were deprotected in 3 mL TAMRA deprotection solution (methanol : *tert*-butylamine : water = 1 : 1 : 2) at 65 °C for 4 h, while all others were deprotected in 3 mL AMA solution (ammonium hydroxide aqueous methylamine = 1 : 1) at 65 °C for 30 min. After precipitation, the oligonucleotide probes were further purified by reversed-phase high-pressure liquid chromatography (HPLC) (ProStar, Varian, Walnut Creek, CA, USA). Oligonucleotide probes with and without diacyllipid groups were purified using a C4 and C18 column, respectively, and the mobile phase was CH_3_CN-TEAA solution. The concentration of probes was quantified using their absorbance at 260 nm, as measured by a Varian Cary 100 UV-vis spectrometer (Agilent Technologies, Santa Clara, CA, USA).

### Real-time PCR measurement

All measurements were performed using a MyiQ™ Single-Color Real-Time PCR Detection System. The temperature was increased from 60 to 80 °C in 1 degree increments and held for 5 min after each increment. Fluorescent signal was measured, and the change in relative fluorescence units (dRFU) was recorded.

### Cell culture

CCRF-CEM (human leukaemia) and HeLa (cervical adenocarcinoma) cell lines were purchased from American Type Culture Collection (Manassas, VA). CCRF-CEM cells were cultured in RPMI-1640 medium (American Type Culture Collection), with 10% fetal bovine serum (Invitrogen, Carlsbad, CA) and HeLa cells were cultured in Dulbecco's modification of Eagle's medium (Fisher Scientific) with 15% fetal bovine serum and 0.5 mg mL^–1^ penicillin–streptomycin (Sigma, St. Louis, MO), both at 37 °C in 5% CO_2_ atmosphere. HeLa cells were grown to 80% confluency for 48 h before incubation with probes.

### Cell lysate preparation

Cells were washed and dispersed in 10 mL of PBS solution. The mixture was lysed with a Branson Sonifier 450 sonicator (200 W) for 1 min, and the resulting cell lysate was stored at 4 °C.

### Cytotoxicity assay

The cytotoxicity of DMFs was evaluated using the CellTiter 96 proliferation assay (Promega, Madison, WI, USA) at 37 °C under 5% CO_2_ atmosphere. A sample of 3000 HeLa cells in 50 μL fresh cell culture medium was seeded into each test well on a 96-well plate. After 12 h, medium was removed from the wells and another 50 μL of fresh medium was added. Then DMFs at the desired concentrations were added to the well. After 48 h treatment, 20 μL of CellTiter 96 reagent was added to the well, and the 96-well plate was subjected to absorption measurement at 490 nm using a VersaMax tunable microplate reader (Molecular Devices, Inc., Sunnyvale, CA, USA). Each experiment has been repeated 5 times and the data are shown as the mean ± the standard deviation. A Student *t*-test was performed and *p* > 0.05 indicated no significance.

### Confocal fluorescence microscopy imaging

HeLa cells were incubated with 1 μM DMFs at 37 °C with 5% CO_2_ for different time lengths, followed by washing twice with PBS to remove free probes. LysoSensor Green (Thermo Fisher) was used for lysosome staining by incubating with cells at 37 °C for 30 min and transferrin from Human Serum, Alexa Fluor 633 Conjugate (ThermoFisher) was incubated with cells for 1 hour. Fluorescence imaging was performed on a Leica TCS SP5 confocal microscope (Leica Microsystems) with a 40× oil-immersion objective. In most cases, the optical slice thickness was adjusted to 0.5 μm. DMFs with TAMRA were excited at 543 nm, and the fluorescence was collected at 570 nm. LysoSensor Green was excited at 443 nm, and the fluorescence was collected at 505 nm. Transferrin was excited at 633 nm, and the fluorescence was collected at 650 nm.

### Fluorescence measurements

A Jobin Yvon fluorometer was used for all fluorescence measurements. For TAMRA, the solutions were excited at 488 nm and scanned from 550 to 650 nm. The anti-photobleaching mode was used for time-dependent measurement.

### Static light scattering

The d*n*/d*c* value of DNA–lipid complex is 0.161 mL g^–1^.^26^ A 2.0 mg sample of DNA micelles was dissolved in 0.7 mL H_2_O/MeOH (90 : 10) and filtered twice before characterization by SEC/Multi-Angle Light Scattering (SEC-MALS) (Agilent Isocratic Pump, Degasser, and Autosampler, SEC-MALS from Wyatt Technology). Light scattering measurements were performed using 3 detectors at 41°, 90° and 139° and a Zimm plot was carried out by ASTRA 6 to obtain the molecular weight of DMFs.

## Conclusions

In summary, using nanoflares as a comparative guideline, this work investigated several basic properties of DNA micelle flares, including aggregation number, critical micelle concentration, dissociation constant and biostability. The aggregation number of DNA–diacyllipid amphiphiles for the lipo-T_60_ micelle is 544, which is comparable to that of nanoflares, but significantly greater than that of DNA block copolymers.^25^ DNA micelle flares have much lower CMC values compared to their polymeric analogues, indicating superior stability in low concentrations, making MBMFs suitable for use as carriers for drugs and genes. In addition, DNA micelle flares also present stronger binding affinities and higher melting temperatures toward their cDNA target relative to DNA probes without diacyllipid conjugation. Most importantly, DNA micelle flares exhibit enhanced biostability in complex biological media, further demonstrating their utility in gene recognition and gene therapy. It was also proven that DMFs could enter the entire cell after 4 hours of incubation with only partial residency in lysosomes. These properties endow DNA micelle flares with the versatility required for bioimaging, drug delivery, and cancer gene therapy, potentially inspiring the functionalization of DNA with multiple oligonucleotides to increase the range of biomedical applications.

## Conflict of interest

The authors declare no competing financial interest.

## Supplementary Material

Supplementary information
