# Supplementary material for: DNA micelle flares: a study of the basic properties that contribute to enhanced stability and binding affinity in complex biological systems
Source: Chem Sci. 2016 May 23;7(9):6041–9. doi: 10.1039/c6sc00066e (PMC5207227; doi:10.1039/c6sc00066e)
Supplement: Supplementary file 1 [file SC-007-C6SC00066E-s001.pdf]

Electronic Supplementary Material (ESI) for Chemical Science.
This journal is © The Royal Society of Chemistry 2016

Supporting Information

Study of the basic properties of DNA micelle flares: enhanced stability and binding affinity

Yanyue Wang,‡ Cuichen Wu,‡ Tao Chen, Hao Sun, Sena Cansiz, Liqin Zhang, Cheng Cui, Weijia Hou, Yuan Wu, Shuo Wan, Ren Cai, Yuan Liu, Brent S. Sumerlin, Xiaobing Zhang* and Weihong Tan*

Center for Research at Bio/Nano Interface, Department of Chemistry and Department of Physiology and Functional Genomics, Health Cancer Center, UF Genetics Institute and McKnight Brain Institute, University of Florida, Gainesville, Florida 32611-7200, United States;

Molecular Science and Biomedicine Laboratory, State Key Laboratory for Chemo/Bio-Sensing and Chemometrics, College of Chemistry and Chemical Engineering, College of Biology, and Collaborative Research Center of Molecular Engineering for Theranostics, Hunan University, Changsha 410082, China

‡Y.W. and C.W. contributed equally to this work.

Supporting Information

Measurement of weight average molecular weight (M_w) using static light scattering (SLS):

A multi-angle light scattering (MALS) instrument can give the concentration-dependent magnitude of the response, termed as the Rayleigh ratio ($R_{\theta,c}$), from photo-detectors at various angles. Combined with known information of a solution, including the refractive index increment of the sample (dn/dc) and sample concentration (c), the molecular weight of a compound can be calculated from the Zimm equation:

$$\frac{KC}{R_{\theta,c}} = \frac{1}{M_w}(1 + \frac{q^2R_g^2}{3} + O(q^4))(1 + 2M_wA_2c + O(c^2))$$

where

$$K = 4\pi^2 n_0^2 (dn/dc)^2 / N_A\lambda^4$$

and

$$q = 4\pi n_0 \sin\left(\frac{\theta}{2}\right)/\lambda$$

with θ the measurement angle, A_2 the second virial coefficient, λ the laser wavelength in a vacuum, N_A the Avogadro's number, n_0 the refractive index of the solvent, and R_g the molecule's radius of gyration.

By building a Zimm plot from a double extrapolation to zero angle and zero concentration from measurements at many angles and many concentrations, the M_w of a certain molecule can be obtained from a reduced Zimm equation:

$$\frac{Kc}{R_{\theta\to 0,c\to 0}} = 1/M_w$$

In the case of DNA micelle flares, the R_θ at zero concentration can be inferred from Figure S1. A Zimm plot was completed by ASTRA 6, which also directly gave the M_w, being 12, 500 g/mol.

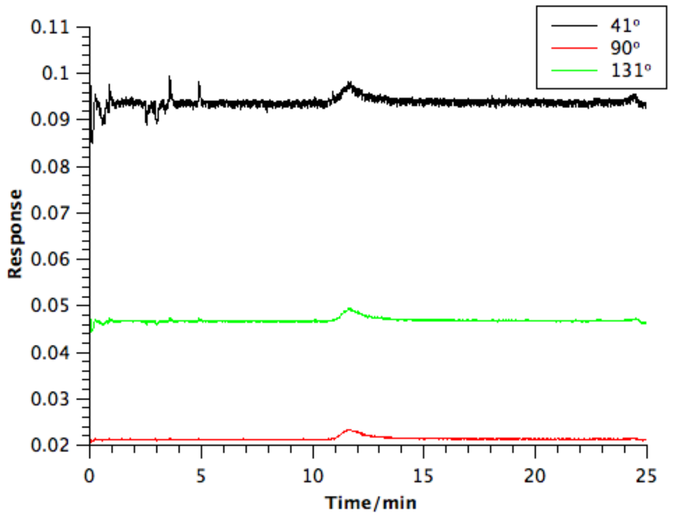

Figure S1. Rayleigh ratio of DMFs measured by detectors at different angles.

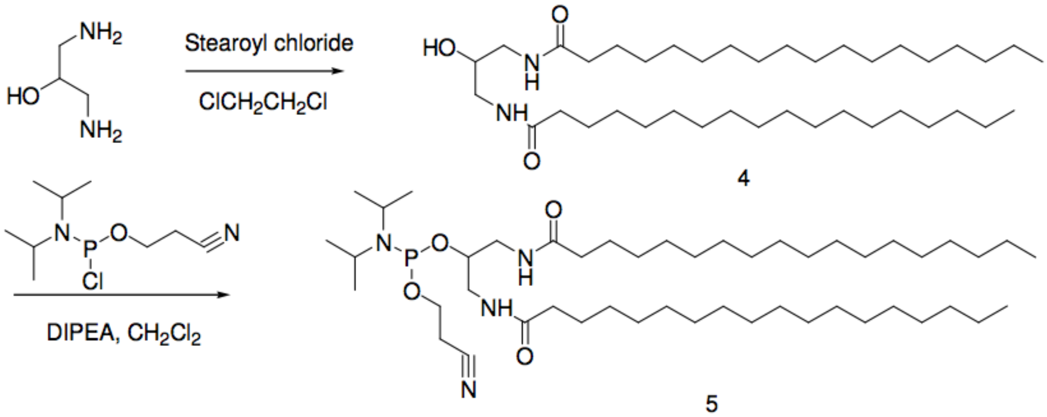

Scheme S1. Detailed synthesis route for lipid phosphoramidite. Mass Spectrometry Analysis (ESI) and HPLC characterization confirmed that the lipid has been successfully coupled to DNA. [1]

Name	Detailed sequence information
MB	5’-Dabcyl-GCGAG TCC CGC CTG TGA CAT GCA TT CTCGC-TAMRA-3’[a]
MBMF	5’-Diacyllipid-Dabcyl-GCGAG TCC CGC CTG TGA CAT GCA TTCTCGC-TAMRA-3’[a]
cDNA	5’-AAT GCA TGT CAC AGG CGG GA-3’
DMF library for endocytosis study	5’- Diacyllipid-NNNNNNNNNNNNNN-NNNNNNNNNNNN –TAMRA-3’

Table S1. The detailed information of DNA sequences.

[a]Underline denotes base pairs in the stem.[2]

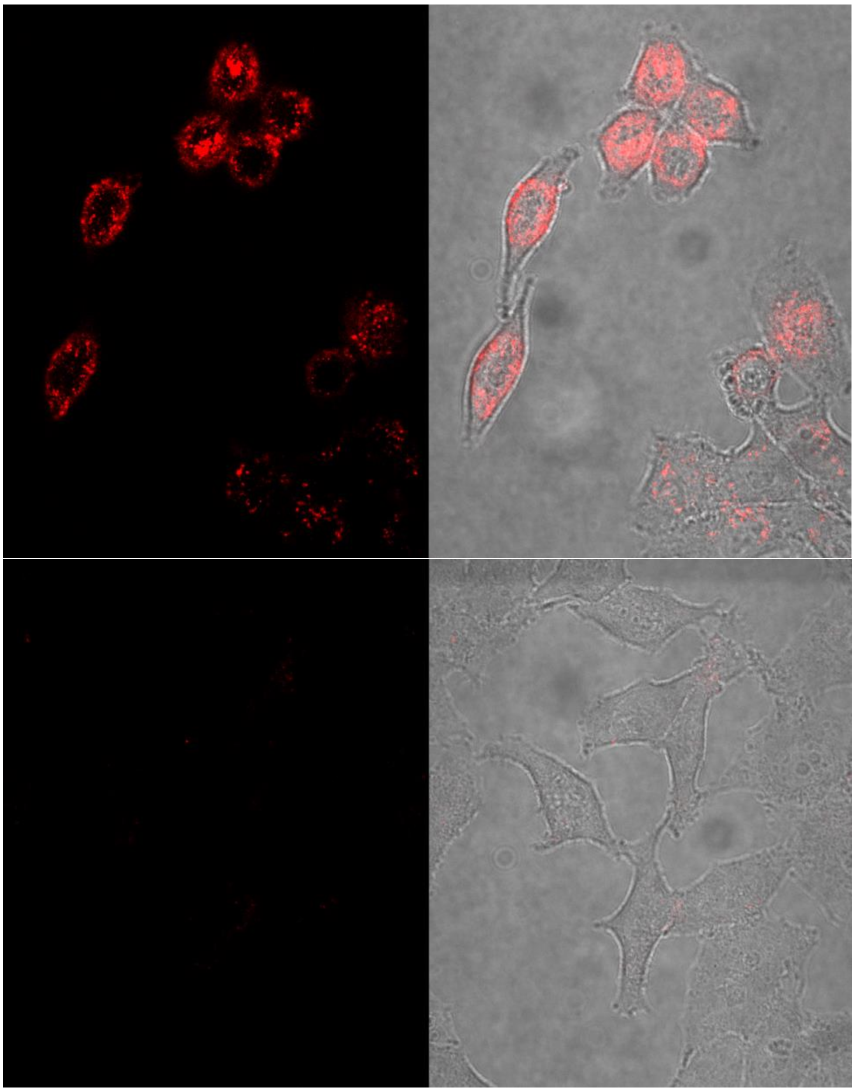

Figure S2. Confocal microscope image of ATP aptamer DMFs incubated with HeLa cells (upper) and library DMFs with HeLa cells (lower). Florescence was observable only with the binding of target molecules.

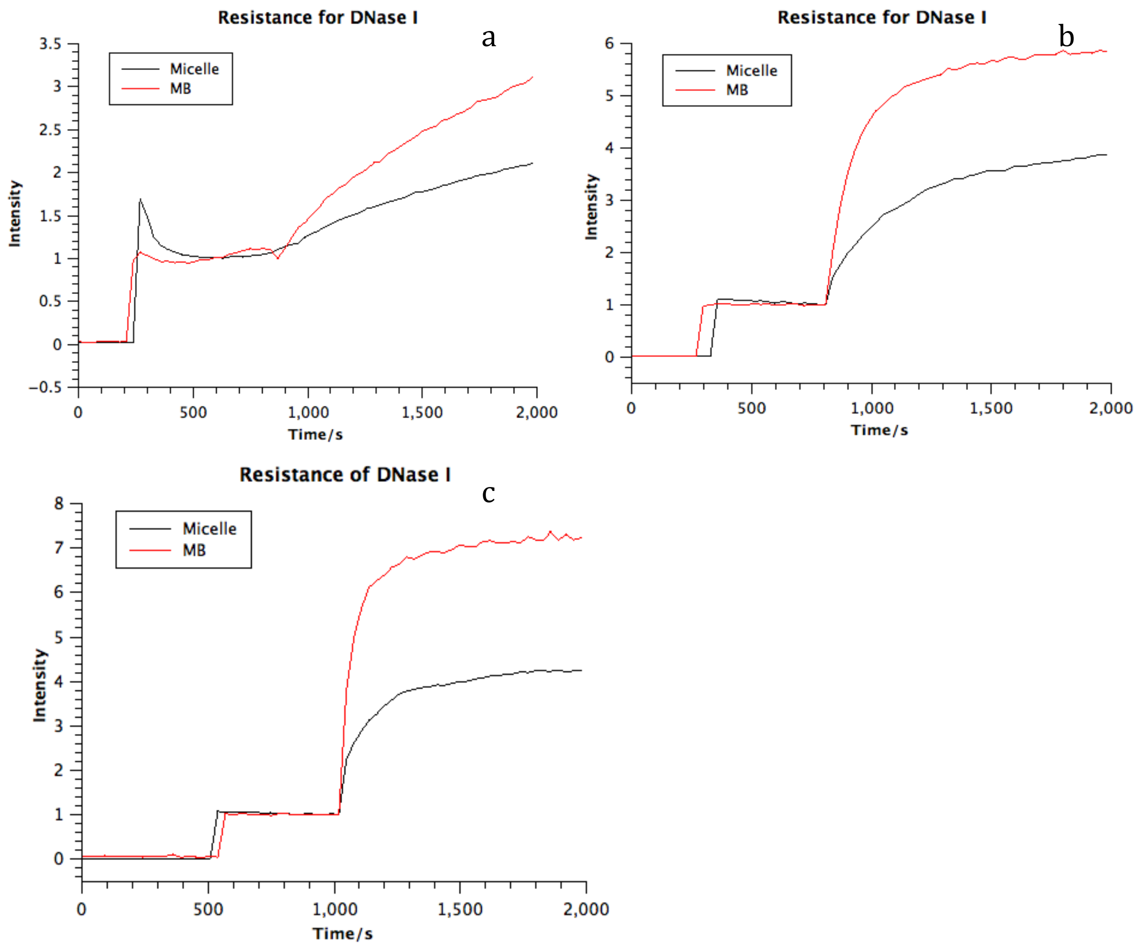

Figure S3. Digestion Curve of MBMFs and MBs with DNase I concentrations of 1U(a), 4U(b) and 10U(c) per 100μL.

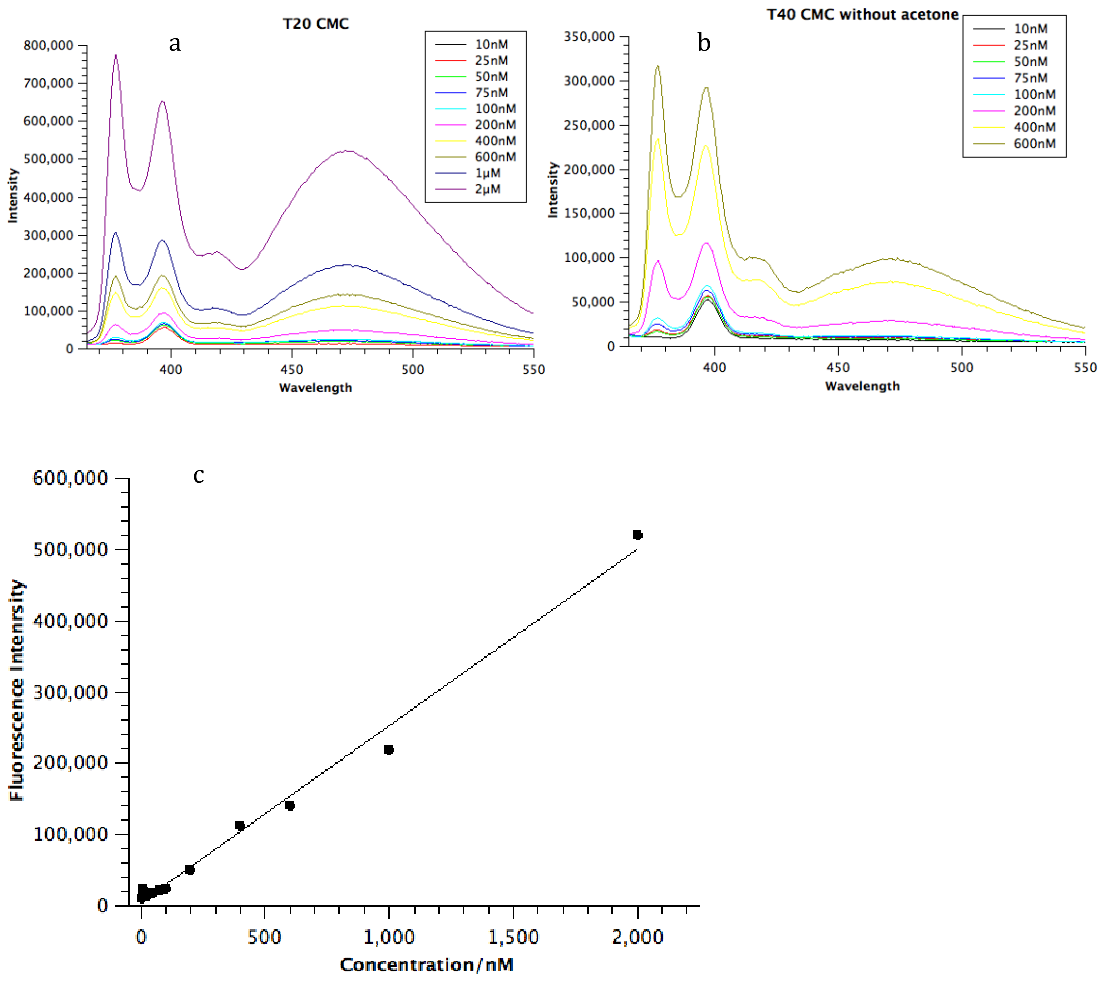

Figure S4. (a-b)CMC curve for DMFs with 20 and 40 T bases. (c) The linear regression of the fluorescence intensity of DMFs according to their concentration.

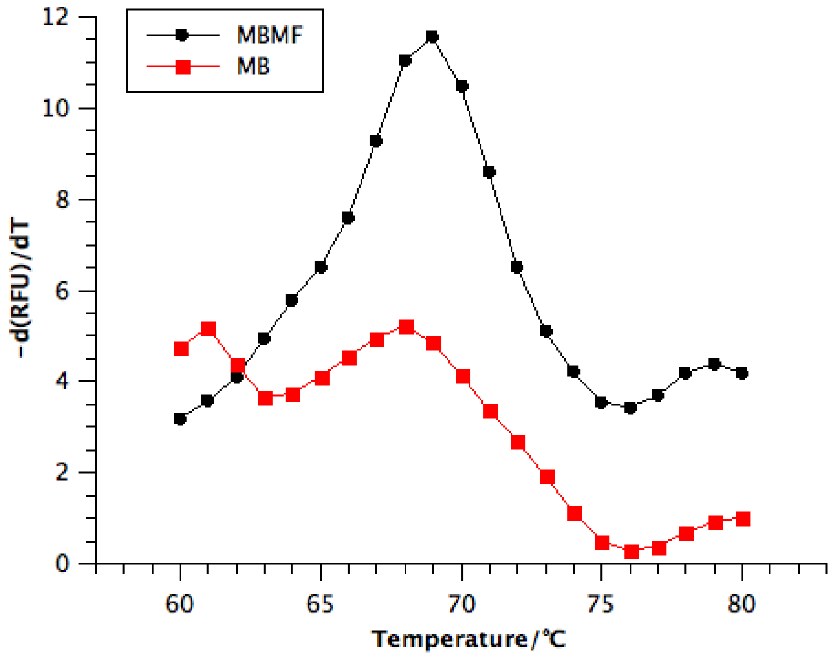

Figure S5. Slope curve for melting transition.

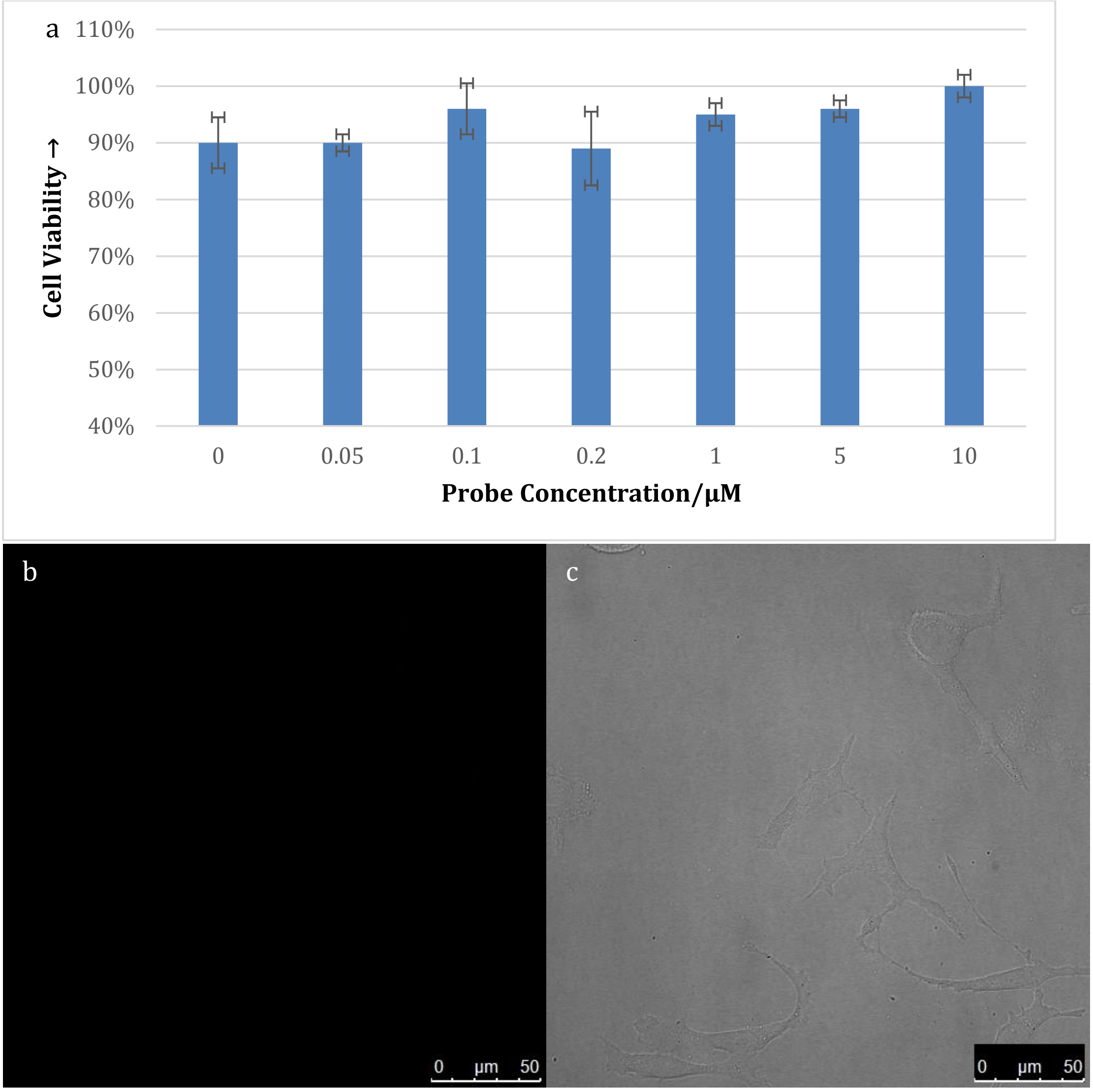

Figure S6. (a)Cell viability of library DMFs. (b-c) Cell internalization of library TAMRA-labeled DNAs and the overlay with cells (c).

Concentrations<br><br>Probe/Dnase I	MB /s^{-1}	MBMF /s^{-1}
200nM/ 1U	0.0035	0.0015
200nM/ 4U	0.0342	0.0176
200nM/ 10U	0.0938	0.0418

Table S2. Initial digestion rates for MBs and MBMFs by DNase I.

References

1. Liu, H., et al., *DNA-Based Micelles: Synthesis, Micellar Properties and Size-Dependent Cell Permeability.* Chemistry – A European Journal, 2010. **16**(12): p. 3791-3797.
2. Chen, T., et al., *DNA Micelle Flares for Intracellular mRNA Imaging and Gene Therapy.* Angewandte Chemie International Edition, 2013. **52**(7): p. 2012-2016.
